# COVID-19 Incidence and Factors Influencing Infection Risk Among People Living With HIV in Türkiye: Is Current Issue the Vaccine Hesitancy-Opposition?

**DOI:** 10.1155/cjid/6767853

**Published:** 2025-08-18

**Authors:** Asuman Inan, Orcun Barkay, Arda Karapınar, Fatma Yılmaz-Karadag, Selman Aktas, Sibel Bolukcu

**Affiliations:** ^1^Department of Infectious Diseases and Clinical Microbiology, Haydarpasa Numune Training and Research Hospital, University of Health Sciences, Istanbul, Türkiye; ^2^Department of Infectious Diseases and Clinical Microbiology, Faculty of Medicine, Erzincan Binali Yıldırım University, Erzincan, Türkiye; ^3^Red Ribbon, Istanbul, Türkiye; ^4^Department of Infectious Diseases and Clinical Microbiology, Sancaktepe Training and Research Hospital, University of Health Sciences, Istanbul, Türkiye; ^5^Department of Biostatistics, Hamidiye Faculty of Medicine, University of Health Sciences, Istanbul, Türkiye; ^6^Department of Infectious Diseases and Clinical Microbiology, Kartal Dr. Lütfi Kırdar Training and Research Hospital, University of Health Sciences, Istanbul, Türkiye

**Keywords:** comorbidities, COVID-19, HIV/AIDS, vaccine hesitancy, vaccine opposition

## Abstract

**Introduction:** As the Coronavirus Disease-2019 (COVID-19) pandemic intersects with the ongoing Human Immunodeficiency Virus/Acquired Immunodeficiency Syndrome (HIV/AIDS) crisis, concerns have emerged regarding the susceptibility of people living with HIV/AIDS (PLWH) to severe outcomes from COVID-19. Despite global efforts to understand the interplay between HIV/AIDS and COVID-19, research on this issue remains limited in Türkiye. This study investigates the incidence of COVID-19 among PLWH in Türkiye and factors influencing infection risk.

**Materials and Methods:** An online survey, conducted from April to June 2023, assessed COVID-19 frequency and risk factors among PLWH aged 18 years and older in Türkiye. Demographic data such as age, gender, educational background, underlying health conditions, vaccination status, and COVID-19 infection history were collected from individuals who voluntarily participated.

**Results:** A total of 354 PLWH from 38 cities in Türkiye participated in the study. The median age was 35.8 (range 18–76); 78% were male and 65.5% were university graduates. The rate of experienced COVID-19 among PLWH was 44.6%. Logistic regression analysis revealed that vaccinated individuals had an 84% lower risk of contracting COVID-19 among PLWH. In sum, 80 participants were not vaccinated in this study; of them, 27.5% identified as vaccine opponents and 25% expressed vaccine hesitancy.

**Discussion:** These findings suggest that vaccination status is the key determinant of COVID-19 susceptibility among young and well-educated PLWH in Türkiye. The notable level of vaccine hesitancy and opposition of PLWH highlights the need for public health initiatives aimed at addressing misinformation and enhancing vaccine confidence.

**Conclusion:** This study underscores the urgent need to address vaccine hesitancy and vaccine opposition among educated PLWH in Türkiye. Amid evolving SARS-CoV-2 variants, vaccination remains paramount in mitigating COVID-19 risks among PLWH. Further research should delve deeper into demographic-specific vaccine concerns to optimize public health strategies and meet the unique needs of PLWH communities.

## 1. Introduction

Currently, approximately 39 million people live with Human Immunodeficiency Virus (HIV); every year, 1.2 million people are newly infected, and 630,000 people die due to HIV/Acquired Immunodeficiency Syndrome (AIDS) and its complications [1]. Since the World Health Organization (WHO) declared the Coronavirus Disease-2019 (COVID-19) a pandemic [2], the Centers for Disease Control and Prevention (CDC) have raised concerns that people living with HIV/AIDS (PLWH) may face a higher risk of COVID-19 complications and mortality compared to the general population [3]. Numerous studies have since been published worldwide on the rates of COVID-19 transmission among PLWH, the disease severity, and the influencing factors in PLWH [4–15]. While some studies report no significant differences in COVID-19 incidence, severity, mortality rates, treatment, or prevention for PLWH [3–7], others indicate that COVID-19 may have a more severe course and higher mortality in PLWH compared to the general population [8–15].

The first COVID-19 case in Türkiye was reported on March 10, 2020. As of November 20, 2023, 17,232,066 people had been affected, with 102,174 deaths recorded [16]. As of the end of November 2023, the Ministry of Health had reported 41,732 HIV/AIDS cases, with 81.5% of cases among men and the majority in the 25–34 age groups [17]. Türkiye is considered a low-endemic country for HIV; however, unlike global trends of decreasing HIV cases, newly infected cases in Türkiye are on the rise [17]. This trend, combined with emerging *Severe Acute Respiratory Syndrome Coronavirus-2* (SARS-CoV-2) variants, raises questions about the consequences of these infections. Few studies have explored this topic [18–22], and no studies on COVID-19 prevalence among PLWH in Türkiye were found in our literature review. This study aimed to investigate COVID-19 rates and influencing factors among PLWH in Türkiye.

## 2. Materials and Methods

### 2.1. Study Design and Participants

This is an online survey study designed to evaluate the frequency of COVID-19 and affecting risk factors in PLWH over the age of 18 in Türkiye. The survey was conducted online through the Red Ribbon Association's website between April 1 and June 23, 2023.

Participants were given identification numbers and were kept anonymous. The survey used in the research consisted of 25 questions related to the aims of the study and was designed using the Google Forms platform. Participants were asked to read and approve the informed consent forms before answering the survey.

Demographic information such as age, gender, educational background, underlying health conditions, vaccination status, and COVID-19 infection history was collected from individuals who voluntarily participated in the study. The “Student” category under occupation was derived from the open-text field in the questionnaire's “Other” option, where respondents were invited to specify their occupation. These responses were then categorized into relevant groups by the study team. The survey also included questions aimed at assessing PLWH's perception of COVID-19 and the preventive measures taken (such as vaccination and personal protective measures). Responses were based on self-reporting, as they were not confirmed by individuals' health records. By conducting this comprehensive survey, the study aimed to obtain valuable insights into the impact of COVID-19 among PLWH with the intention of informing future public health interventions and providing a deeper understanding of the factors influencing COVID-19 outcomes in this specific population.

  Definition of Vaccination: Uptake of the full primary series.
  Definition of Vaccine Hesitancy: Delay in acceptance or refusal of vaccines despite the availability of vaccination services [23].
  Definition of Vaccine Opposition: Active refusal or resistance to receive vaccines despite the availability of vaccination services which may stem from a range of factors including personal beliefs, distrust in vaccine safety and effectiveness, misinformation, or socio-political views [24].

### 2.2. Statistical Analysis

Data were analyzed using SPSS version 25.0 and R software. Qualitative variables were expressed as frequency and percentage, and quantitative variables that did not meet the assumptions of normality (as assessed by the Shapiro–Wilk test) were summarized with medians and ranges (minimum–maximum). The Mann–Whitney U test was employed to compare two independent groups for non-normally distributed variables. Chi-square tests were applied to examine associations between categorical variables.

For multivariable analysis, binary logistic regression was performed to identify independent predictors of COVID-19 positivity among PLWH. Variables considered clinically relevant and those with a *p* value < 0.20 in univariate analyses were entered into the model. The reference categories were as follows: Female (gender), full-time job (occupation), college education (education), < 500 USD (income), and vaccinated (for vaccine status). Model fit was assessed using the Hosmer–Lemeshow test (*p*=0.979), and explained variance was calculated via Cox and Snell *R*^2^ (0.169) and Nagelkerke *R*^2^ (0.222).

A sensitivity analysis was conducted by switching the gender reference category (i.e., male as the reference), and results were consistent in terms of statistical nonsignificance.

### 2.3. Ethics Approval

This study was approved by the Ethics Committee of Health Sciences University, Sancaktepe Training and Research Hospital (Approval number: SBU-SSIVEAH-15).

## 3. Results

A total of 354 PLWH from 38 cities in Türkiye participated in the study. Among these individuals, 158 (44.63%) stated that they had experienced COVID-19 at least once with a positive test result.

Of all participants, 78.2% (*n* = 277) were male. The median age was 35.8 (range 18–76), 65.5% had received education at the university level, and all patients were receiving antiretroviral therapy.

The average age among individuals who had and had not COVID-19 was 35.5 and 36, respectively, indicating no statistically significant difference. There were slightly more female individuals in the COVID-19 positive group (14.6%) than in the negative group (10.7%), though this was not statistically significant (*p*=0.567). Similarly, there were no significant differences between the COVID-19 positive and negative groups in terms of occupation, education, and income variables (*p*=0.185, *p*=0.552, and *p*=0.286, respectively) (Table 1). It was noteworthy that most of the patients had university degrees in both the COVID-19 positive and negative groups. The proportion of patients with more than one comorbidity was higher in the COVID-19 positive group (7.6%) than the negative group (6.6%); however, the difference was not statistically significant (*p*=0.725).

The association of various conditions such as comorbidities, COVID-19 history, and vaccination status results are shown in Table 2. Totally, 80 (22.6%) of the cases were not vaccinated. While 38.6% of those who had COVID-19 were unvaccinated, 9.7% of those who did not have COVID-19 were unvaccinated. Of those who were unvaccinated, 22 individuals (27.5%) identified as vaccine opponents (stating ‘I am against vaccines and do not believe in COVID-19'), while 20 individuals (25%) expressed vaccine hesitancy (stating ‘I am afraid of the side effects of vaccines and believe they are ineffective'). Overall, 42 individuals, constituting 52.5% of the unvaccinated group and 11.8% of all participants, reported being either vaccine opponents or experiencing vaccine hesitancy. As a result of the analysis, statistical significance was found between COVID-19 vaccination status and COVID-19 status (*p* < 0.001). All other variables did not show a significant difference with COVID-19 status (Table 2).

Among the 158 individuals who had tested positive for COVID-19, 10 (6.3%) required hospitalization. One individual (0.6%) was admitted to the intensive care unit (ICU). Thirty participants (18.9%) used antiviral drugs such as hydroxychloroquine, favipiravir, or molnupiravir. Reinfection was reported by 33 individuals (20.8%). Of these, five were unvaccinated, and one of them required hospitalization. The ICU-admitted patient was also unvaccinated and reported disbelief in COVID-19 as a real disease.

33 (20.8%) individuals had reported experiencing the illness two or more times. Among them, including one who required hospitalization, five were currently unvaccinated. Additionally, one individual who reported a positive test result and was hospitalized in intensive care had also not been vaccinated and expressed disbelief in COVID-19 disease.

In the logistic regression model, male gender was used as the reference category. Neither female (OR = 1.498; 95% CI: 0.705–3.184; *p*=0.293) nor other gender identity (OR = 0.850; 95% CI: 0.360–2.008; *p*=0.711) was significantly associated with COVID-19 positivity. While the wide confidence intervals observed for gender may limit the interpretability of sex-specific odds ratios, nonsignificant findings remained consistent across sensitivity analyses, supporting the robustness of the model. This consistency was also observed when the reference category for gender was changed.

As a result of binary logistic regression analysis, statistical significance was found only in the vaccination status variable, and it was observed that those who were vaccinated had an 84% lower risk of contracting COVID-19 (Table 3).

## 4. Discussion

Studies have been focused on how the COVID-19 pandemic has affected HIV-positive people. Many studies about co-infection situations have shown that people with HIV do not appear to be more seriously impacted by SARS-CoV-2 than those who do not have HIV [3–7]. Our findings align with these studies. It is well known that with advancements in early detection and treatment options, the PLWH is aging, leading to an increase in the number of comorbidities they experience [1]. The relationship of comorbidities with COVID-19 infection has been studied in many studies [25–29]. For instance, Richardson et al. found that comorbidities such as hypertension, diabetes, and obesity are risk factors for COVID-19 infection [25]. These studies show that comorbidities may affect the course of COVID-19 infection, and individuals with comorbidities may have more serious disease outcomes [25, 29]. Similar observations have been reported in studies examining the impact of multiple comorbidities on COVID-19 outcomes in various populations [30]. In our study, although the number of patients with more than one comorbidity was higher in the COVID-19 positive group, the difference was not significantly meaningful. We think that this is due to the fact that the average age of the patients participating in our study was much lower than in other studies.

A widespread belief is that the sole goal of the PLWH population in general should not be solely focused on suppressing viral replication. There is a shift towards improving quality of life and adopting a new “90%” target [31, 32]. Considering this goal, access to preventive health care services such as vaccinations during the pandemic period for individuals living with HIV/AIDS will have positive effects on improving their quality of life and life expectancy. In our country, health authorities have anticipated the vulnerability of the PLWH population during the COVID-19 pandemic, leading to initiatives such as encouraging people to stay home and prioritizing access to vaccines [33]. Additionally, free access to vaccines increases vaccine uptake, especially in developing countries like ours. However, unfortunately, we are in a period where vaccine hesitancy and opposition continue and increase worldwide. Similar opposing views also exist in Türkiye.

It was noteworthy that approximately 22% of individuals remained unvaccinated in our study. When our findings about the rate of vaccine opposition are compared to global and regional data, the vaccine uptake rate in our cohort aligns with some mid-range estimates. For example, a study conducted in Nigeria reported a lower vaccine uptake rate of 57.7% among PLWH, with vaccine hesitancy [34]. Similarly, in South Africa, a vaccine uptake rate of 61% was reported, where hesitancy was linked to similar concerns [14]. In contrast, studies conducted in Canada and Europe have shown higher uptake rates [35, 36]. This discrepancy highlights the importance of health care access and public health interventions in mitigating vaccine opposition.

Although the vaccine uptake rate in Türkiye was relatively high compared to low-resource settings, the persistence of vaccine opposition among a significant portion of the population underscores the need for targeted interventions [33]. Addressing misinformation and vaccine concerns should remain a priority to further increase uptake and protect this vulnerable population from future COVID-19 variants. A cross-sectional survey of community-based research from Türkiye showed that 45.3% of participants reported vaccine hesitancy for COVID-19 [37].

In a study conducted among HIV-negative individuals in our country, the reasons behind vaccine opposition during the COVID-19 period were investigated. The majority of these individuals cited the absence of chronic illness as a reason for their opposition. Some also stated either their own or their close contacts' successful recovery from COVID-19, leading them to believe that they did not need the COVID-19 vaccine [38]. Another study conducted in Türkiye revealed that individuals experiencing vaccine refusal/hesitancy tended to acquire information about vaccines from the media at higher rates compared to those who received vaccine-related information from health care professionals. The study also demonstrated higher levels of vaccine hesitancy among those concerned about vaccine side effects and those who did not believe the vaccine was the most effective way to overcome the pandemic [39]. In a multicenter study among COVID-19 vaccine-naïve PLWH in Nigeria, participants highlighted that the top reasons for vaccine hesitancy were concerns about rapid vaccine development and fear of potential side effects [34]. In another study conducted in China, a comparison was made between PLWH individuals and the general healthy population, revealing a higher rate of vaccine opposition among PLWH individuals. The primary reasons attributed to this were concerns about the effectiveness and safety of the vaccine [40].

The majority of the participants in our study had received higher education, yet we lack sufficient data to determine whether their reasons for not being vaccinated stem from inadequate access to accurate information on the internet or interruptions in general health care services during the pandemic period. Gürbüz and Aydın demonstrated a decrease in vaccine confidence among university students, of whom the participants showed a high rate of “yes” responses to questions like “Can alternative medicine methods be used instead of vaccines?” or “Do you think the vaccine will cause infertility?” [41]. Considering the higher proportion of young and highly educated individuals in our study, it is speculated that they might have chosen not to get vaccinated due to similar concerns as those within the general population. We also desired to underline the importance of vaccination again, as we still have unvaccinated highly educated individuals, although we know that COVID-19 vaccinations for patients with HIV are safe and effective, according to declarations from international organizations [42].

Our study has some limitations, the most significant one is being a survey-based study, and the data are collected based on individuals' self-reports. The survey being accessible via a civil society organization's website might have led to the participation of individuals with a certain level of age, education and socio-cultural background, thereby not fully representing all PLWH in our country. Additionally, the self-reported nature of responses means it is unknown whether individuals who claimed not to have had COVID-19 experienced it asymptomatically or with very mild symptoms.

## 5. Conclusion

Our study suggests that among young and well-educated PLWH, there are no significant differences in susceptibility to COVID-19, with the exception of vaccination status, which remained a significant protective factor. Although a substantial proportion of unvaccinated individuals had higher levels of education and income, these factors were not independently associated with vaccine hesitancy or opposition in multivariable analysis.

The most notable finding is the high rate of vaccine hesitancy and opposition observed in this relatively well-educated and higher-income sample. While these results are important, they should be interpreted with caution and viewed as hypothesis-generating due to the limitations of our sampling method—online, self-selected, and predominantly composed of young male participants with higher education levels—which may restrict generalizability and influence observed trends.

As new SARS-CoV-2 variants continue to circulate globally, vaccination remains the most effective and cost-efficient strategy for preventing COVID-19. Therefore, addressing vaccine-related concerns through targeted public health interventions is crucial for reducing COVID-19 risk in vulnerable populations such as PLWH.

Finally, while studies conducted in different regions and populations have yielded mixed findings regarding the impact of HIV on COVID-19 outcomes, vaccine hesitancy and opposition stand out as critical concerns [43]. Future research should explore the complex, underlying factors influencing vaccine attitudes in PLWH through more representative sampling strategies and in-depth qualitative approaches to guide tailored health policies in Türkiye and beyond.

## Figures and Tables

**Table 1:** Demographic characteristics of the patients.

		COVID-19		Statistic	*P*
		+ *n* = 158 (45%)	− n = 196 (55%)		
Gender	Male	121 (76.6%)	156 (79.6%)	1.206	0.547^a^
	Female	23 (14.6%)	21 (10.7%)		
	Other	14 (8.8%)	19 (9.7%)		

Occupation	Full-time	117 (74.2%)	125 (63.8%)	4.82	0.185^a^
	Retired	15 (9.5%)	29 (14.8%)		
	Unemployed	15 (9.5%)	21 (10.7%)		
	Student	11 (7%)	21 (10.7%)		

Education	College	30 (19%)	46 (23.5%)	1.189	0.552^a^
	High school	20 (12.7%)	26 (13.3%)		
	University	108 (68.4%)	124 (63.2%)		

Income (USD)	< 500	42 (26.6%)	67 (34.2%)	2.501	0.286^a^
	> 800	62 (39.2%)	66 (33.7%)		
	500–799	54 (34.2%)	63 (32.1%)		

Age		35.5 (19–68)	36 (18–76)	15,014	0.623^b^

Abbreviation: USD, United States Dollars.

^a^Chi-square test.

^b^Mann–Whitney U test.

**Table 2:** Association of various parameters and COVID-19 test results.

		COVID-19		*χ* ^2^	*p*
		+ *n* = 158 (45%)	− *n* = 196 (55%)		
Vaccination status	**+**	97 (61.4%)	177 (90.3%)	41.81	< 0.001
	−	61 (38.6%)	19 (9.7%)		

No other diseases	**+**	145 (74%)	114 (72%)	0.149	0.700
	−	51 (26%)	44 (28%)		

Diabetes	**+**	8 (5.1%)	11 (5.6%)	0.052	0.82
	−	150 (94.9%)	185 (94.4%)		

Overweight	**+**	17 (10.8%)	13 (6.6%)	1.921	0.166
	−	141 (89.2%)	183 (93.4%)		

Hypertension	**+**	14 (8.9%)	16 (8.2%)	0.055	0.815
	−	144 (91.1%)	180 (91.8%)		

Cardiovascular disease	**+**	6 (3.8%)	11 (5.6%)	0.63	0.427
	−	152 (96.2%)	185 (94.4%)		

Lung disease	**+**	5 (3.2%)	7 (3.6%)	0.044	0.833
	−	153 (96.8%)	189 (96.4%)		

Cancer	**+**	3 (1.9%)	2 (1%)	0.485	0.486
	−	155 (98.1%)	194 (99%)		

Chronic renal failure	**+**	1 (0.6%)	4 (2%)	1.245	0.264
	−	157 (99.4%)	192 (98%)		

Cardiac failure	**+**	1 (0.6%)	1 (0.5%)	0.023	0.878
	−	157 (99.4%)	195 (99.5%)		

More than one comorbidity	**+**	12 (7.6%)	13 (6.6%)	0.123	0.725
	−	146 (92.4%)	183 (93.4%)		

**Table 3:** Examining the effects of variables with binary logistic regression analysis.

	*β*	S.E.	*p*	Exp (B)	95% C.I. for exp (B)	
					Lower	Upper
Age	−0.015	0.017	0.395	0.985	0.953	1.019
Gender			0.520			
Female	−0.404	0.385	0.293	1.498	0.705	3.184
Other	−0.162	0.438	0.711	0.850	0.360	2.008
Vaccine	−2.025	0.317	**0 < 0.001**	0.132	0.071	0.246
Job			0.300			
Retired	−0.461	0.564	0.413	0.63	0.209	1.904
Unemployed	−0.55	0.475	0.246	0.577	0.227	1.463
Student	−0.795	0.525	0.13	0.451	0.161	1.263
Education			0.244			
High-school	0.512	0.458	0.264	1.668	0.679	4.096
University	0.542	0.34	0.111	1.719	0.882	3.351
Income (USD)			0.567			
> 800	0.297	0.394	0.451	1.346	0.622	2.913
500–799	0.376	0.353	0.287	1.457	0.729	2.912
No other disease	−0.556	0.703	0.429	0.574	0.145	2.277
Diabetes	−0.355	0.811	0.662	0.701	0.143	3.435
Overweight	0.091	0.751	0.903	1.095	0.252	4.77
Hypertension	0.047	0.749	0.950	1.048	0.241	4.553
Cardiovascular disease	−0.738	0.834	0.376	0.478	0.093	2.452
Lung diseases	−1.445	1.655	0.383	0.236	0.009	6.04
Cancer	1.23	1.19	0.301	3.423	0.332	35.267
Chronic renal failure	−1.701	1.421	0.231	0.183	0.011	2.956
Cardiac failure	−0.873	2.146	0.684	0.418	0.006	28.007
Comorbidity	1.29	1.149	0.261	3.634	0.382	34.531
Constant	−0.704	2.434	0.772	0.495		

*Note:* Hosmer–Lemeshow test p:0.979; Cox and Snell R:0.169; Nagelkerke *R*^2^: 0.222; Overall percentage: 68.9; Reference category: Simple (first), Reference categories: Gender–Female; Occupation–Full-time; Education–College; Income–< 500 USD; Vaccination–Vaccinated. Bold values indicate statistically significant results (*p* < 0.05).

Abbreviations: S.E., standard error; C.I., confidence interval; USD, United States Dollars.

## Data Availability

The data that support the findings of this study are available from the corresponding author upon reasonable request.
